# Runs of Homozygosity and NetView analyses provide new insight into the genome-wide diversity and admixture of three German cattle breeds

**DOI:** 10.1371/journal.pone.0225847

**Published:** 2019-12-04

**Authors:** Sowah Addo, Stefanie Klingel, Dirk Hinrichs, Georg Thaller

**Affiliations:** 1 Institute of Animal Breeding and Husbandry, Kiel University, Kiel, Germany; 2 Department of Animal Breeding, University of Kassel, Witzenhausen, Germany; 3 Center for Rare and Endangered Domestic Animals, Arche Warder, Warder, Germany; National Cheng Kung University, TAIWAN

## Abstract

Angler (RVA) and Red-and-White dual-purpose (RDN) cattle were in the past decades crossed with influential Red Holstein (RH) sires. However, genome-wide diversity studies in these breeds are lacking. The objective of the present study was to elucidate the genome-wide diversity and population structure of the three German cattle breeds. Using 40,851 single nucleotide polymorphism markers scored in 337 individuals, runs of homozygosity (ROH) were analysed in each breed. Clustering and a high-resolution network visualisation analyses were performed on an extended dataset that included 11 additional (outgroup) breeds. Genetic diversity levels were high with observed heterozygosity above 0.35 in all three breeds. Only RVA had a recent past effective population size (*N*_*e*_) estimate above 100 at 5 generations ago. ROH length distribution followed a similar pattern across breeds and the majority of ROH were found in the length class of >5 to 10 Mb. Estimates of average inbreeding calculated from ROH (F_ROH_) were 0.021 (RVA), 0.045 (RDN) and 0.053 (RH). Moderate to high positive correlations were found between F_ROH_ and pedigree inbreeding (F_PED_) and between F_ROH_ and inbreeding derived from the excess of homozygosity (F_HOM_), while the intercept of the regression of F_ROH_ on F_PED_ was above zero. The population structure analysis showed strong evidence of admixture between RVA and RH. Introgression of RDN with RH genes was minimally detected and for the first time, the study uncovered Norwegian Red Cattle ancestry in RVA. Highly heterogeneous genetic background was found for RVA and RH and as expected, the breeds of the extended dataset effectively differentiated mostly based on geographical origin, validating our findings. The results of this study confirm the impact of RH sires on RVA and RDN populations. Furthermore, a close monitoring is suggested to curb further reduction of *N*_*e*_ in the breeds.

## Introduction

Following their domestication, cattle became adapted to specific environments into which they were introduced by their human capturers. The complexity of their origin, coupled with both artificial and natural selection culminated into the development of numerous breeds that exhibit variable phenotypes linked to different local conditions across regions of the world [1,2]. In the past few decades, however, factors including advances in breeding methods, industrialisation of cattle husbandry and the globalization of human society have resulted in the development of specialised high yielding breeds, which are often used at the expense of local breeds [1,3]. This calls for urgent actions at regional, national and international levels to save local breeds from extinction.

In Schleswig-Holstein in the northern part of Germany, two local cattle breeds namely, Angler (RVA) and Red-and-White dual-purpose (RDN) cattle are regionally well acknowledged and kept in commercial enterprises. RVA is popular for its high milk fat percentage and though a dual-purpose breed, kept primarily for milk production. It has contributed to the genetics of many local red breeds of central and eastern European countries and in the Baltic region [4,5]. Similarly, RDN was used to improve the performance of the Belgian native Red-and-White breed some decades ago [6]. The performance RVA and RDN in terms of milk yield falls below that of Red Holstein (RH) cattle in the same region. Therefore, breeders attempted to improve milk yield of the local breeds by the introduction of genes from high yielding breeds. Bennewitz and Meuwissen [7] highlighted the introgression of RVA with genetics of other breeds (e.g. RH and Swedish Red), which happened in the 1960s. For RDN, a pedigree of the breed typically harbours RH genes reaching about 25% [8]. Like other local breeds, RVA and RDN are small in numbers and their respective herdbook numbers are declining. To our knowledge, genetic diversity studies in these breeds are very limited. For instance, a pedigree-based assessment of genetic diversity in the RVA and RDN populations revealed some influential RH sires being key ancestors of these populations [9]. The influence of same sires in both populations makes it interesting to study genome-wide diversity across these three breeds. In an independent study [10], a close relationship (genome based kinship coefficient = 0.039) was found between RVA and RH although principal component analysis put the two breeds apart.

Genome-based genetic diversity studies are nowadays the method of choice given the recent advances in SNP chips technology and the developments of advanced computational methodologies in population genetics [11–13]. Medium to high density SNP panel has been used especially in cattle [14–17] and to a lesser extent in sheep [18–20], goats [21,22] and horses [23,24]. In most of these studies, principal component analysis (PCA) and the model-based admixture analysis [25] were effectively used to infer population structure between breeds. More recently, a high-definition network visualisation tool (NetView) was introduced [26] and further developed [27] to investigate population differentiation and produce fine-scale population structures without prior knowledge of individual ancestry. For studies involving within breed diversity, an inbreeding concept based on runs of homozygosity (ROH) is gaining wider acceptance [28]. These methodologies provide new approaches that can be applied in local breeds to guide conservation decision. Therefore, the objective of the current study was to use various methods in the assessment of whole-genome diversity of three German cattle breeds (RVA, RDN and RH) and furthermore, investigate admixture and gene flow events amongst them. The study also investigated the usefulness of ROH in these breeds. Findings of the research will provide insight into the composition and diversity of the breeds.

## Material and methods

### Data and data preparation

Genotyping data on 338 animals including 169 RVA, 69 RDN and 100 RH individuals were obtained from the official computation centre responsible for breeding value estimation (vit, Verden, Germany) for the analyses. The vit also provided pedigree data on RVA and RDN. All animals were genotyped with the Illumina BovineSNP50 BeadChip: 207 with chip type v2 featuring 54,609 SNPs and 131 with v1 (54,001 SNPs). The data were merged in PLINK [29] after the removal of all non-autosomal and non-annotated SNPs. The merged dataset, consisting of 51,105 SNPs, was filtered to keep individuals with 95% of their SNPs called and SNPs with 95% call rate across all samples. SNPs with minor allele frequencies (MAF) lower than 5% or that deviate from Hardy Weinberg expectation (*P* value ≤ 0.0001) were excluded. After filtering, 337 individuals remained, all having the same 40,851 SNPs with an average distance of 60.496 kb (minimum, 0.065 kb; maximum, 937.787 kb) and a mean r^2^ value of 0.186 between adjacent SNPs (S1 Table). Using quality filtered data, the “—genome” function in PLINK [29] was used to derive genomic relationship coefficients between all individuals for each breed. Subsequently, from the pairs of highly related individuals (relationship coefficient > 0.30), one animal was randomly discarded to ensure an appropriate representation of the diversity of each breed. This step was necessary to minimize within breed clustering in population structure analysis. Finally, 106 RVA, 47 RDN and 88 RH individuals remained and were used for all calculations except in the detection of ROH where all 337 individuals were used. Besides the original data for this study as describes, genotype information on 274 individuals available publicly at the “Web-Interfaced next generation Database dedicated to genetic Diversity Exploration” (WIDDE) [30] were included as outgroup data in the population structure analyses. These cover 11 different breeds: 10 genotyped with the Illumina BovineSNP50 BeadChip and 1 (i.e. Angus) with the Illumina HD chip (Table 1). The outgroup data were pruned applying previously mentioned filtering criteria and were subsequently merged with the quality controlled original dataset. The resulting dataset had 32,566 SNPs on 515 individuals.

**Table 1:** Description and source of publicly available outgroup data on 274 individuals across 11 breeds.

Breed	Acronym	Land of Origin	Number	Original data source
**Abondance**	ABO	France	22	[1]
**Angus**	ANG	Scotland	42	Illumina HD project
**Baoule**	BAO	Burkina Faso	28	[31]
**Brown Swiss**	BSW	Switzerland	24	[32]
**Charolais**	CHA	France	20	[1]
**Guernsey**	GNS	Channel Islands	21	[32]
**Holstein**	HOL	Northern Europe	30	[1]
**Jersey**	JER	Channel Islands	21	[1]
**Montbeliarde**	MON	France	30	[1]
**Norwegian Red Cattle**	NRC	Norway	21	[32]
**Red Angus**	RGU	Scotland	15	[32]

### Heterozygosity and genomic effective population size

Observed heterozygosity (H_o_) was estimated for each breed using the “—het” command in PLINK [29]. This was calculated as a difference between the number of homozygotes and the number of non-missing genotypes, expressed as a proportion of non-missing genotypes. The recent past demographic history of each breed was studied by estimating effective population sizes (*N*_*e*_) through linkage disequilibrium (LD) applying the SNeP package [33]. SNeP predicts *N*_*e*_ following Corbin *et al*. [34]:

$$N_{T(t)}={\left(4f(c_{t})\right)}^{-1}(E{\left[r_{adj}^{2}|c_{t}\right]}^{-1}-\propto )$$

where *N*_*T*(*t*)_ is the effective population size *t* generations ago calculated as *t* = (2*f*(*c_t_*))^−1^ [35], *c*_*t*_ is the recombination rate for a specific physical distance between SNPs estimated using Sved and Feldman approximation [36], $r_{adj}^{2}$ is the LD value adjusted for sample size and α is a correction for the occurrence of mutations. Default settings of the programme were used apart from a specification of sample size and a setting of the maximum distance between SNPs to 10 Mb. The chosen distance allowed the estimation of *Ne* related to 5 generations back (Ne_5Gen_), which was considered as the most recent estimate following Deniskova *et al*. [19]. For much recent generations, LD-based *Ne* tends to be unreliable [34]. The extent of LD decay in each breed was analysed from PLINK [29] generated LD values which were calculated for markers on the same chromosome that are less than 2 Mb apart.

### Runs of homozygosity and inbreeding

For each of the 337 individual, ROH were detected using PLINK [29] and by applying a sliding window of 50 SNPs, allowing one possible heterozygous genotype, up to two missing genotypes, a minimum SNP density of 1 SNP every 100 kb and a maximum gap of 1 Mb between consecutive homozygous SNPs. Next, the abundance and size distribution of ROH were investigated. ROH were categorized based on their physical length into 1 to 5 Mb, >5 to 10 Mb, >10 to 15 Mb, >15 to 20 Mb, >20 to 25 Mb, >25 to 30 Mb and >30 Mb as in previous studies [21,22]. Furthermore, the total number of ROH and the sum of all ROH segments were calculated for all animals per breed and per ROH length category. Genomic inbreeding coefficient based on ROH (F_ROH_) was calculated for each animal following McQuillan *et al*. [28]., i.e. *F_ROH_* = ∑*L_ROH_/L_AUTO_*, where ∑*L*_𝑅𝑂𝐻_ refers to the total length of all ROH in the genome of an individual and *L*_*AUTO*_ is the length of the autosomal genome coverage by SNPs in the analysis (2,499,219 kb). Following Mészáros *et al*. [37], F_ROH_ was calculated for three different minimum lengths of ROH including 4, 8 and 16 Mb representing F_ROH>4_, F_ROH>8_ and F_ROH>16,_ respectively. An alternative genomic inbreeding coefficient (F_HOM_) was calculated for each animal based on the excess in the observed number of homozygous genotypes within an individual relative to the mean number of homozygous genotypes expected under random mating [38]. The observed and expected number of homozygous genotypes were obtained using the “—het” PLINK [29] command. Pedigree-based inbreeding coefficients (F_PED_) were calculated for 155 RVA and 65 RDN genotyped individuals with 3,375 and 2,204 available records of ancestors, respectively. F_PED_ was calculated following the algorithm of Meuwissen and Luo [39] as implemented in Pedig [40]. Average pedigree completeness at the fifth parental generation was 88% and 89% for the 155 and 65 individuals, respectively. Finally, F_ROH,_ F_HOM_ and F_PED_ were compared using linear regression and Pearson’s correlation coefficient (*r*_*p*_).

### Population structure

To characterise the population structure, LD pruning was performed on the quality filtered data using the “—indep 50 5 2” PLINK [29] command. Only 19,971 SNPs remained for this analysis. In the first step, PLINK [29] was used to generate genome-wide pairwise identity-by-state (IBS) distances between individuals as well as eigenvectors on which basis the relationships between breeds were studied using PCA. Secondly, the programme Admixture [25], which uses a model-based clustering algorithm, was used to determine the optimal number of k clusters (i.e. the most likely number of contributing populations), and to describe individual ancestry in terms of these clusters. Using the “cv-flag” of the programme, a 20-fold cross-validation procedure was performed for a range of k between 1 and 40, and the k with the lowest cross-validation error was considered as the optimal number of clusters for the data. Cluster assignments ranging from k = 2 to k = 20 were graphically presented using Pophelper [41]. Thirdly, a NetView analysis [26] which uses an unsupervised clustering method known as Super Paramagnetic Clustering (Spc) was applied to detect fine-scale population structures based on genetic distances. Network construction and the clustering of individuals were implemented using the R version of NetView [26,27]. The programme requires a specification of the maximum number of nearest neighbours (K-NN) an individual can have. K-NN was set to 10, 35 and 100 to investigate the characteristics of both fine- and large-scale genetic structures. Following clustering, population networks were visualised using Cytoscape v3 [42]. Finally, admixture results at the optimal k clusters were included in the network construction such that node colour represents individual level of admixture at the selected number of clusters.

## Results

### Genetic diversity parameters

**Fig 1:**
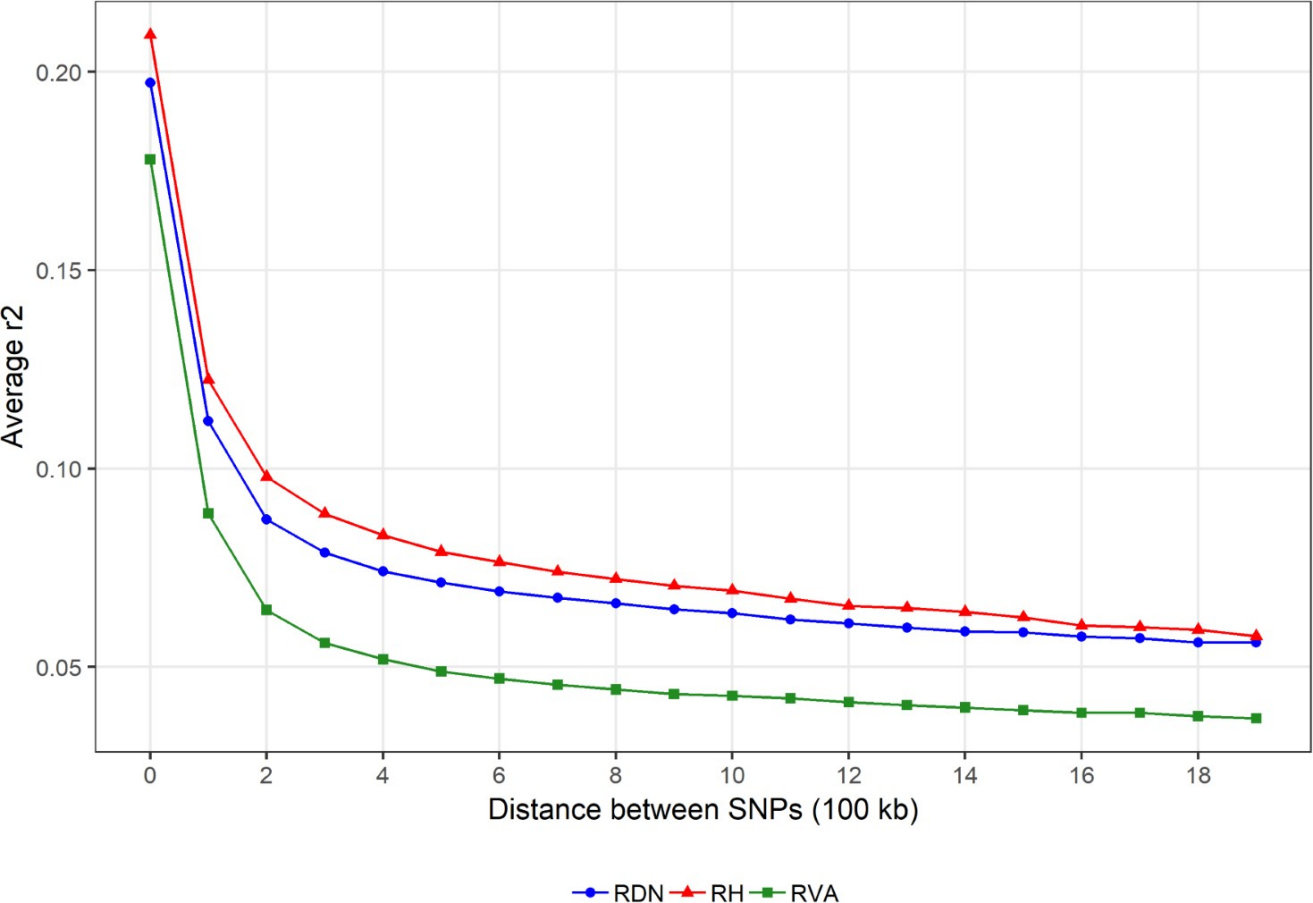
Average linkage disequilibrium (LD) decay in Angler (RVA), Red-and-White dual-purpose (RDN) and Red Holstein (RH).

**Fig 2:**
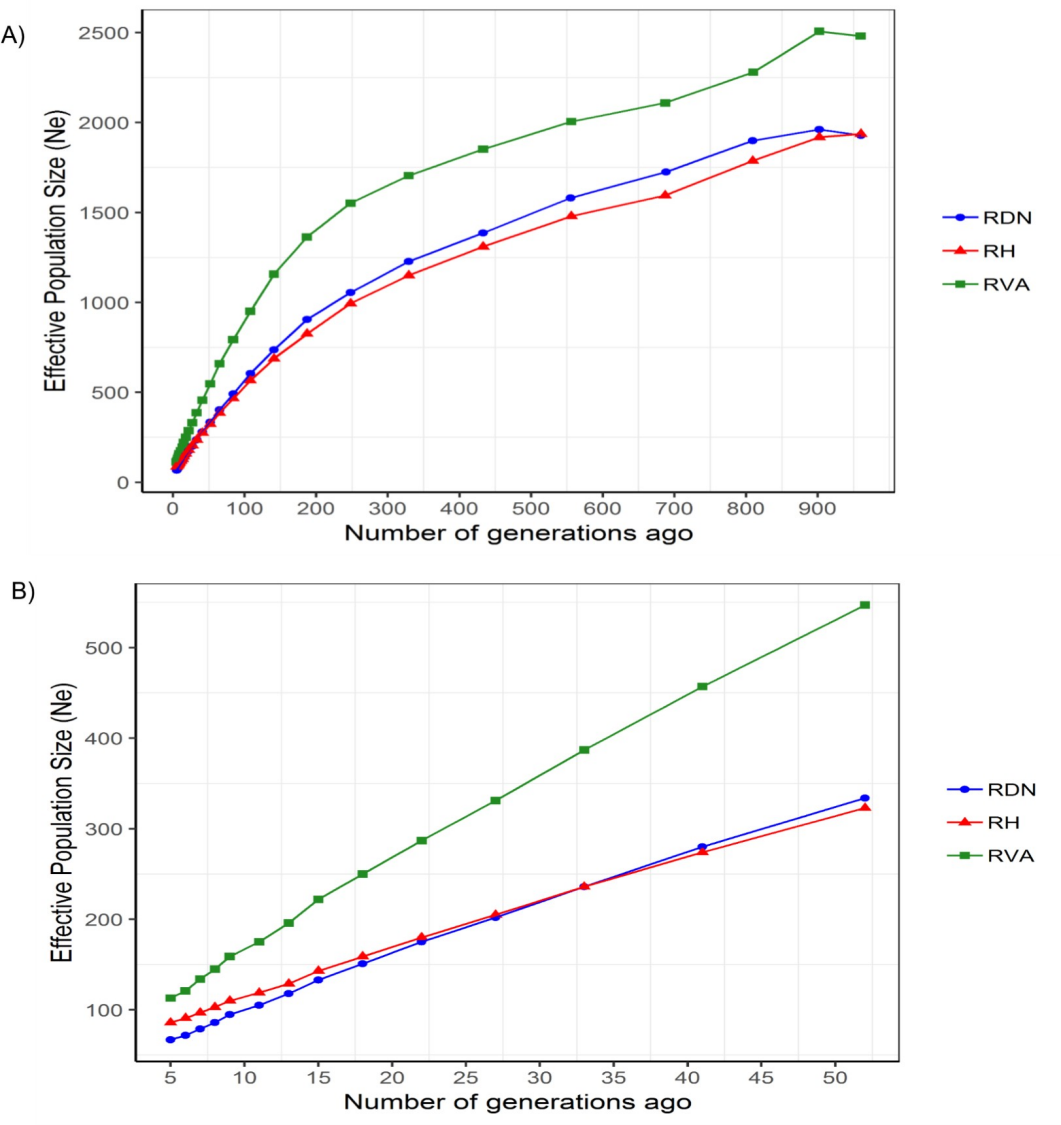
Trends in LD based effective population size for Angler (RVA), Red-and-White dual-purpose (RDN) and Red Holstein (RH). A) Across 1000 generation and B) across 52 generations ago.

Table 2 summarises genetic diversity parameters including relationship coefficients, heterozygosity and effective population size (estimates). Average genomic relationship coefficients (Ø gRel) were 0.028 in RH, 0.031 in RVA and 0.033 in RDN. Estimates of H_o_ were high in all breeds and reached 0.374 for RVA. Average LD decreased along growing inter-marker distances, with a slight variation across breeds (Fig 1). RVA (green) displayed the fastest LD decay while RH (red) showed the slowest. The estimate of Ne_5Gen_ was largest in RVA (113) and smallest in RDN (67). For all breeds, *N*_*e*_ declined over time (Fig 2A). RVA maintained the largest *N*_*e*_ across all generations. For several generations, *N*_*e*_ trend in RDN was slightly above that in RH until about 30 generation ago when the trajectories became the same and the opposite happened then after (Fig 2B).

**Table 2:** Number of animals (N), average genomic relationship (Ø gRel), average observed heterozygosity (H_o_) and most recent effective population size (Ne_5Gen_) for Angler (RVA), Red-and-White dual-purpose (RDN) and Red Holstein (RH).

Breed	N	Ø gRel (±SD)	H_o_ (±SD)	Ne_5Gen_
**RVA**	106	0.031 (± 0.048)	0.374 (± 0.008)	113
**RDN**	47	0.033 (± 0.048)	0.356 (± 0.013)	67
**RH**	88	0.028 (± 0.046)	0.363 (± 0.010)	86

### Runs of homozygosity and inbreeding

**Fig 3:**
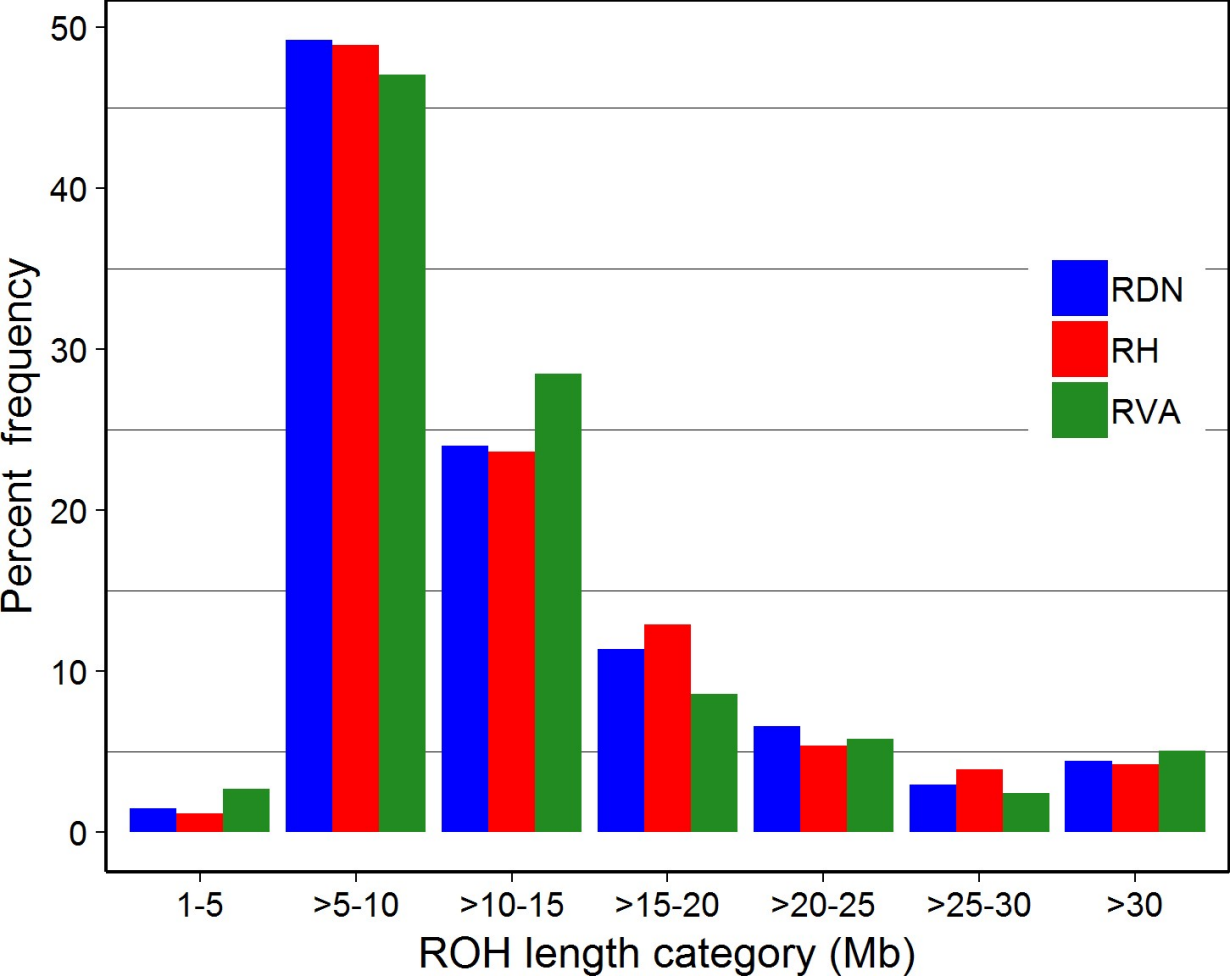
Frequency distribution of the number of ROH in different length classes for Angler (RVA), Red-and-White dual-purpose (RDN) and Red Holstein (RH).

The number and length of ROH segments differed considerably among animals and across breeds (Table 3). ROH segments were identified in all but 14 RVA individuals. Averaging the total number of ROH segments over the number of animals with identified ROH, RH had the highest number of ROH segments (10.25), whilst RVA had the lowest number (4.61). RH and RDN had greater proportion of their autosome, 0.53 (132.51 Mb) and 0.45 (112.91 Mb), respectively, covered with ROH. The proportion of autosomal genome length covered by ROH in RVA was 0.23 and comparatively small. Variation existed in the distribution of the various ROH length classes, but a common pattern was followed across breeds (Fig 3). The majority of ROH segments (~47 to 49%) were found in the length class >5 to 10 Mb for all breeds. Within the second highest represented length class, >10 to 15 Mb, the proportion of ROH was highest in RVA (28%) and about the same in RDN and RH (~24%). Longer ROH segments (>30 Mb) were very few (~5%) but the proportion was even smaller for length class 1 to 5 Mb.

**Table 3:** Number of animals with and without identified ROH, total and average number of ROH per breed and average sum of ROH segment lengths for Angler (RVA), Red-and-White dual-purpose (RDN) and Red Holstein (RH).

Breed	No. of animal without ROH	No. of animal with ROH	Total No. of ROH	Avg. No. of ROH segments (min-max)	Avg. sum of ROH segment lengths in Mb (min-max)
**RVA**	14	154	710	4.61 (1–13)	58.16 (5.91–187.67)
**RDN**	0	69	608	8.81 (1–20)	112.91 (5.09–281.13)
**RH**	0	100	1,025	10.25 (3–20)	132.51 (23.26–298.49)

Regardless of the minimum length of ROH threshold or method of computation, inbreeding was generally lower in RVA, intermediate in RDN and highest in RH (Table 4). When the minimum length of ROH was set to 4 Mb, average F_ROH_ varied from 2.1% (RVA) to 5.3% (RH). The estimates varied from 1.8% to 4.4% and 0.9% to 2.3% for minimum length setting of 8 and 16 Mb, respectively. F_ROH_ within breed also varied (see standard deviations) with values reaching over 11% in some RDN and RH individuals. Overall, F_ROH_ decreased with increased minimum ROH length threshold. Negative values were obtained for F_HOM_ in most individuals. Estimation of F_PED_ was only possible for RVA and RDN since pedigree records were not available for the RH genotypes. The completeness of pedigree in RVA and RDN are provided in S1 Fig.

**Table 4:** Mean inbreeding coefficients calculated from ROH with minimum length of 4 (F_ROH>4_), 8 (F_ROH>8_), and 16 (F_ROH>16_) Mb, from the excess of homozygosity (F_HOM_) and from pedigree (F_PED_) for Angler (RVA), Red-and-White dual-purpose (RDN) and Red Holstein (RH).

Breed	F_ROH>4_ Mean (±SD)	F_ROH>8_ Mean (±SD)	F_ROH>16_ Mean (±SD)	F_HOM_ Mean (±SD)	F_PED_ Mean (±SD)
**RVA**	0.021 (± 0.017)	0.018 (± 0.016)	0.009 (± 0.012)	-0.015 (±0.026)	0.013 (± 0.012)
**RDN**	0.045 (± 0.028)	0.038 (± 0.025)	0.020 (± 0.019)	-0.017 (± 0.038)	0.023 (± 0.020)
**RH**	0.053 (± 0.024)	0.044 (± 0.024)	0.023 (± 0.020)	-0.010 (± 0.029)	NA

**Fig 4:**
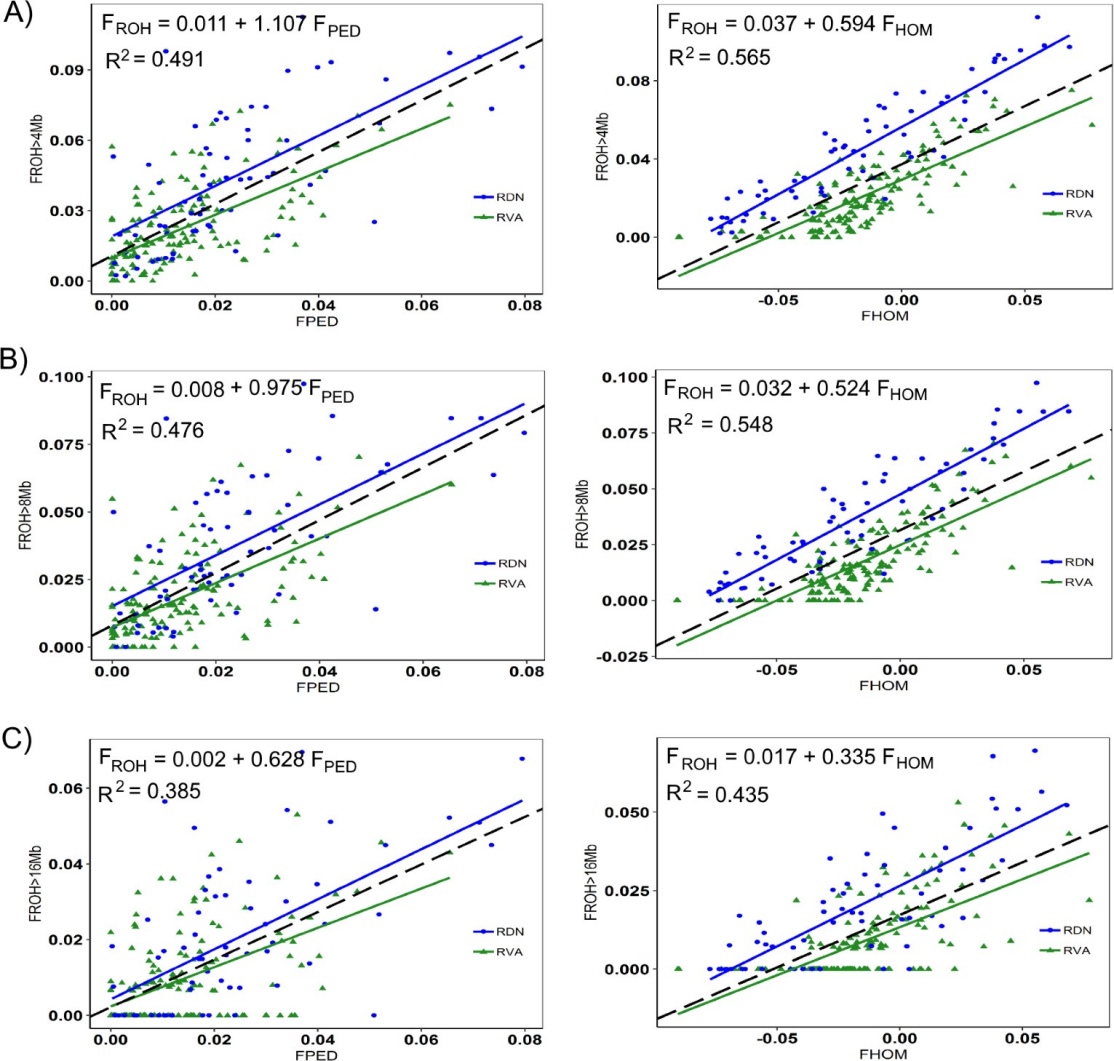
**Regression plot of FROH_>4Mb_ (A), F_ROH>8Mb_ (B) and F_ROH>16Mb_ (C) on F_PED_ (left) and on F_HOM_ (right) for Angler (green) and Red-and-White dual-purpose (blue).** The broken line (black) is a regression line for both breeds and corresponds to the regression equation presented.

The pedigree-based average inbreeding coefficients estimated were generally lower than those computed from ROH. In Fig 4, the linear regression plots of F_ROH_ on F_PED_ (left) and F_ROH_ on F_HOM_ (right) for RVA and RDN are provided for the different ROH length thresholds (A, B and C). Within and across breeds, moderate to high correlations significantly different from 0 (p-value < 2.2e-16) were found for all comparisons. Between F_ROH_ and F_PED,_ *r*_*p*_ was strongest for the 4 Mb threshold (0.701, R^2^ = 0.491), followed by 8 Mb (0.690, R^2^ = 0.476) and then 16 Mb (0.620, R^2^ = 0.385). The correlations between the two genomic inbreeding measures (i.e. F_ROH_ vs. F_HOM_) were slightly stronger and followed the afore-mentioned pattern, thus 4 Mb (0.752, R^2^ = 0.565), followed by 8 Mb (0.740, R^2^ = 0.548) and then 16 Mb (0.660, R^2^ = 0.435). An investigation of the relationship between F_HOM_ and F_PED_ showed a comparatively weaker correlation (0.511, R^2^ = 0.261) between the two inbreeding measures, although significant at p-value = 5.246e-16 (S2 Fig). The intercept of the regression of F_ROH_ on F_PED_ was high (0.011) at the minimum ROH length threshold of 4 Mb and close to 0 (0.002) when the minimum length was increased to 16 Mb.

### Population structure

**Fig 5:**
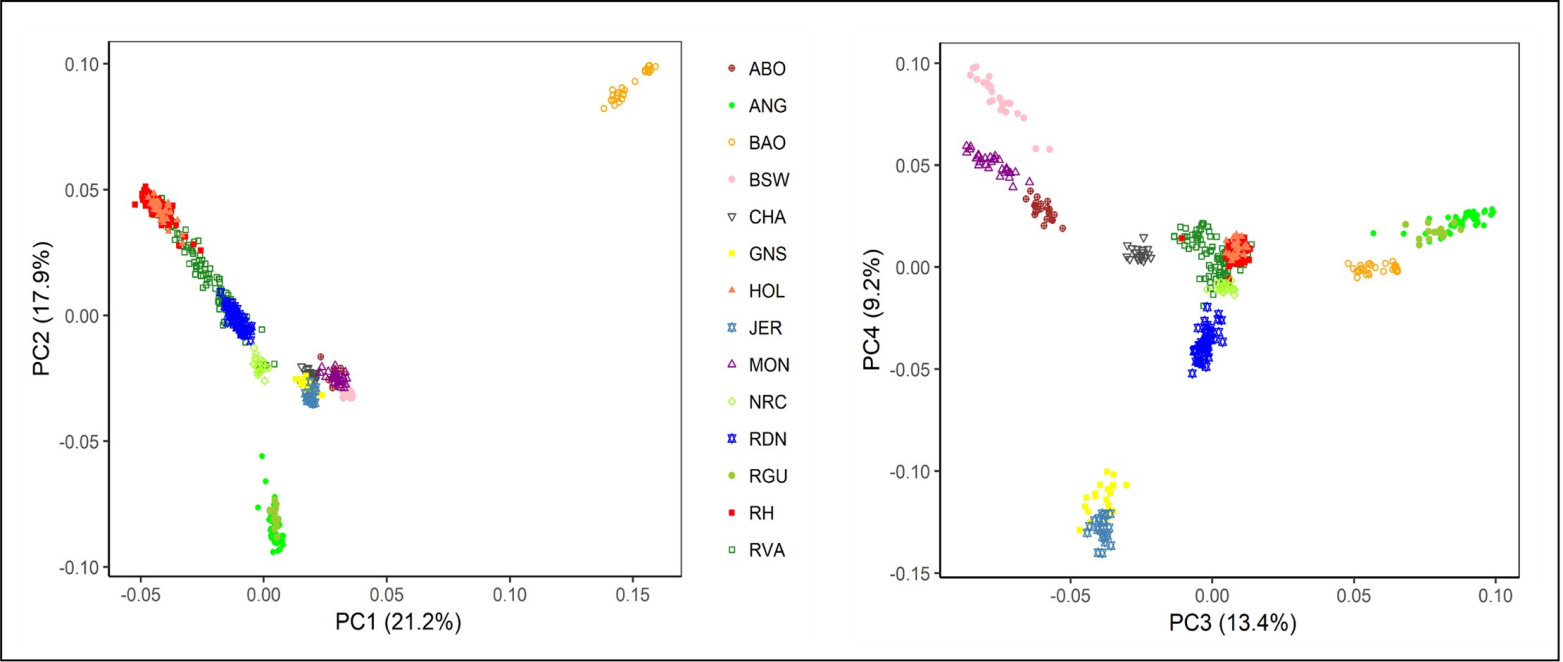
Principal component analysis of 14 cattle breeds distinguished by shape and colour. Based on 19,971 SNPs, the first four components together accounted for 61.7% of variation in the data.

The first principal component (PC1) explained 21.2% while the second (PC2), third (PC3) and fourth (PC4) explained 17.9%, 13.4% and 9.2%, respectively of total variation in the data (Fig 5). Contrasting PC1 vs. PC2, the West African breed (BAO) was clearly separated from the rest of the breeds all of which originated from Europe. Among the European breeds, some clustering occurred along geographical lines. The Northern European breeds (HOL, RH, RVA, RDN and NRC) formed what seemed like an interlocking cluster clearly separated from Scottish breeds (ANG and RGU) on one hand and on the other hand, the remaining breeds originating from France (ABO, CHA and MON), Channel Islands (JER and GNS) and Switzerland (BSW). A plot of PC3 vs. PC4 resulted in a clear separation of the Channel Island, French and Swiss breeds. Within the French breeds, there was a close proximity between ABO and MON while CHA was distinctly separated. The closeness between JER and GNS was maintained similar to the situation in ANG and RGU. For the Northern breeds, only RDN was partially separated from the rest of the group. The HOL samples cluster completely with samples from RH and the two overlaid a fraction of genotypes from RVA.

**Fig 6:**
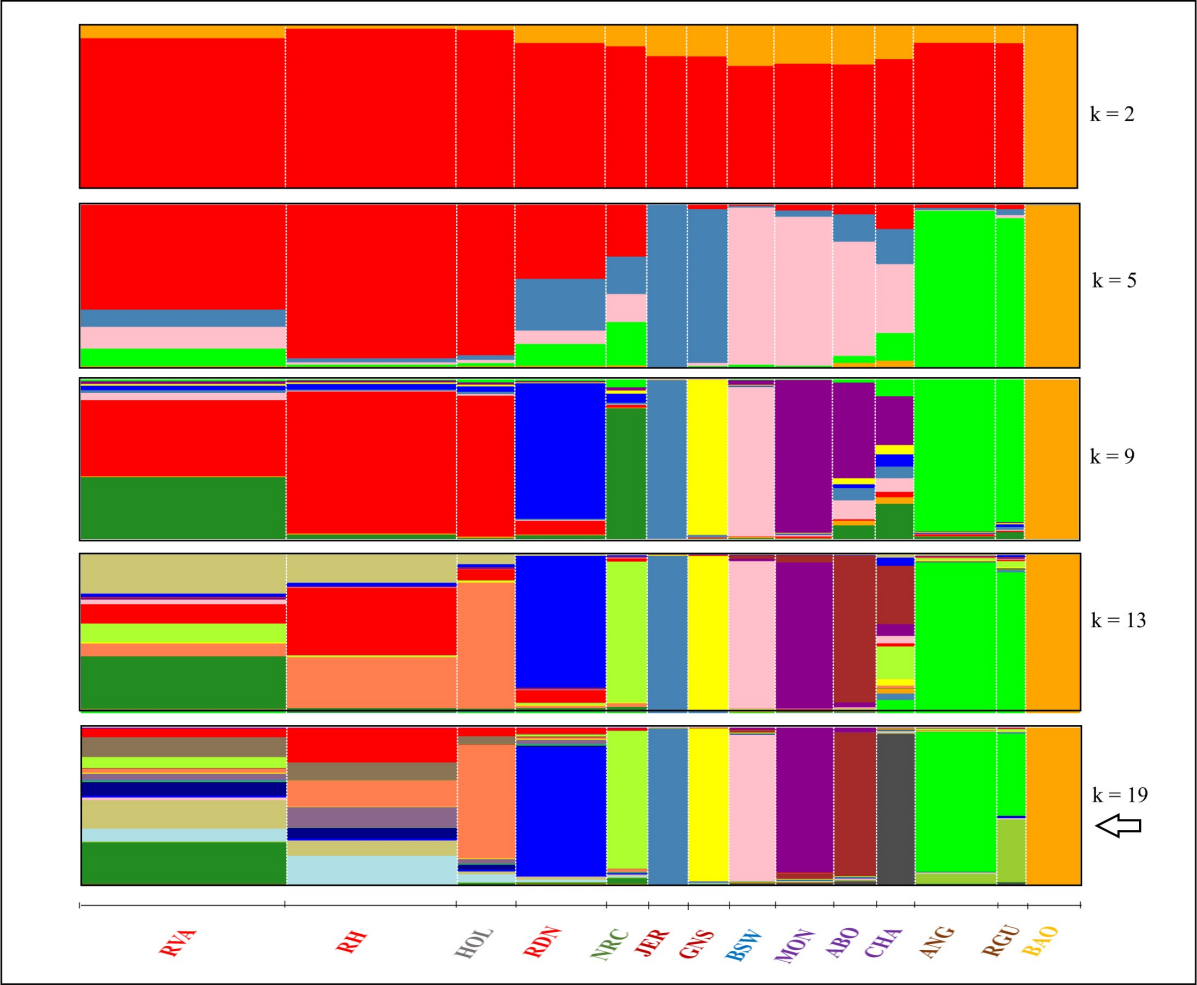
Admixture graphs for k = 2, 5, 9, 13 and 19 with the optimal cluster level marked with an arrow. The amount of a colour in a cluster reflects a breed’s proportion of genetic variation originating from that colour. Breed names are coloured according to geographical origin: Germany (red), Northern Europe (grey), Norway (green), Channel Islands (dark red), Switzerland (blue), France (purple), Scotland (brown) and Burkina Faso (orange).

An optimal number of k = 19 was determined as the most probable number of inferred clusters adjudging from the lowest cross-validation error estimate (S3 Fig). Admixture graphs of the cluster analysis for k values being 2, 5, 9, 13 and 19 are shown in Fig 6 (see S4 Fig for k values ranging from 2 to 20). The analysis at k = 2 revealed a partial differentiation of the African breed (BAO) from the European ones (predominantly red background). As the value of k increased, BAO maintained its unique ancestry while the European breeds differentiated mostly based on geographical origin. Consistent with the findings of PC1 vs. PC2, the admixture results at k = 5, showed a near identical genetic background for JER and GNS (steel blue) and for ANG and RGU (green). Additionally, the Northern breeds (RVA, RH, HOL, RDN and NRC) showed varying degrees of admixture (predominantly red) in a similar fashion as the Swiss and French breeds (predominantly pink). At k = 9, the French breeds (magenta) differentiated almost entirely from BSW; GNS (yellow) differentiated from JER; and RDN (blue) and NRC (forest green) showed higher degree of distinction from the rest of the Northern breeds. Here, NRC shared more of its ancestry with RVA while genetic background of RH and HOL was still almost identical. At k = 13, ABO and CHA differentiated from MON and some degree of dissimilarity of ancestor fractions was observed between RH and HOL for the first time. At the optimal number of k clusters (k = 19), all three French breeds were effectively differentiated although specks of admixture among them was evident. At this cluster level, there was persistency of the varying levels of admixture among the Northern breeds. RVA showed the highest level of admixture with RH and HOL and besides, these breeds, especially RVA and RH had highly mixed genetic background.

**Fig 7:**
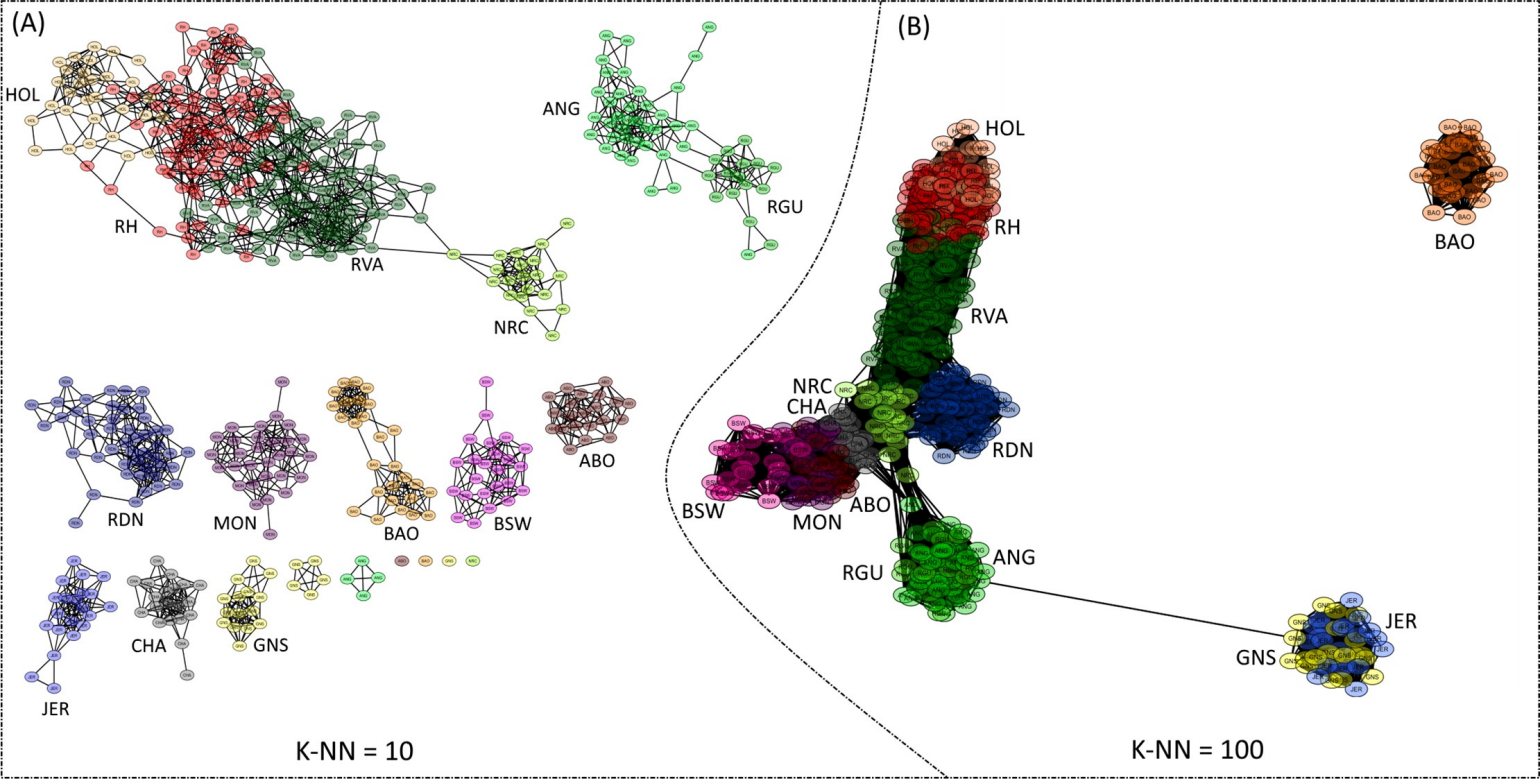
Visualization of high-resolution population networks for 14 cattle breeds. K-NN = 10 (A) investigated small-scale structures, K-NN = 100 (B) targeted large-scale structures and breeds are distinguished by node colour.

**Fig 8:**
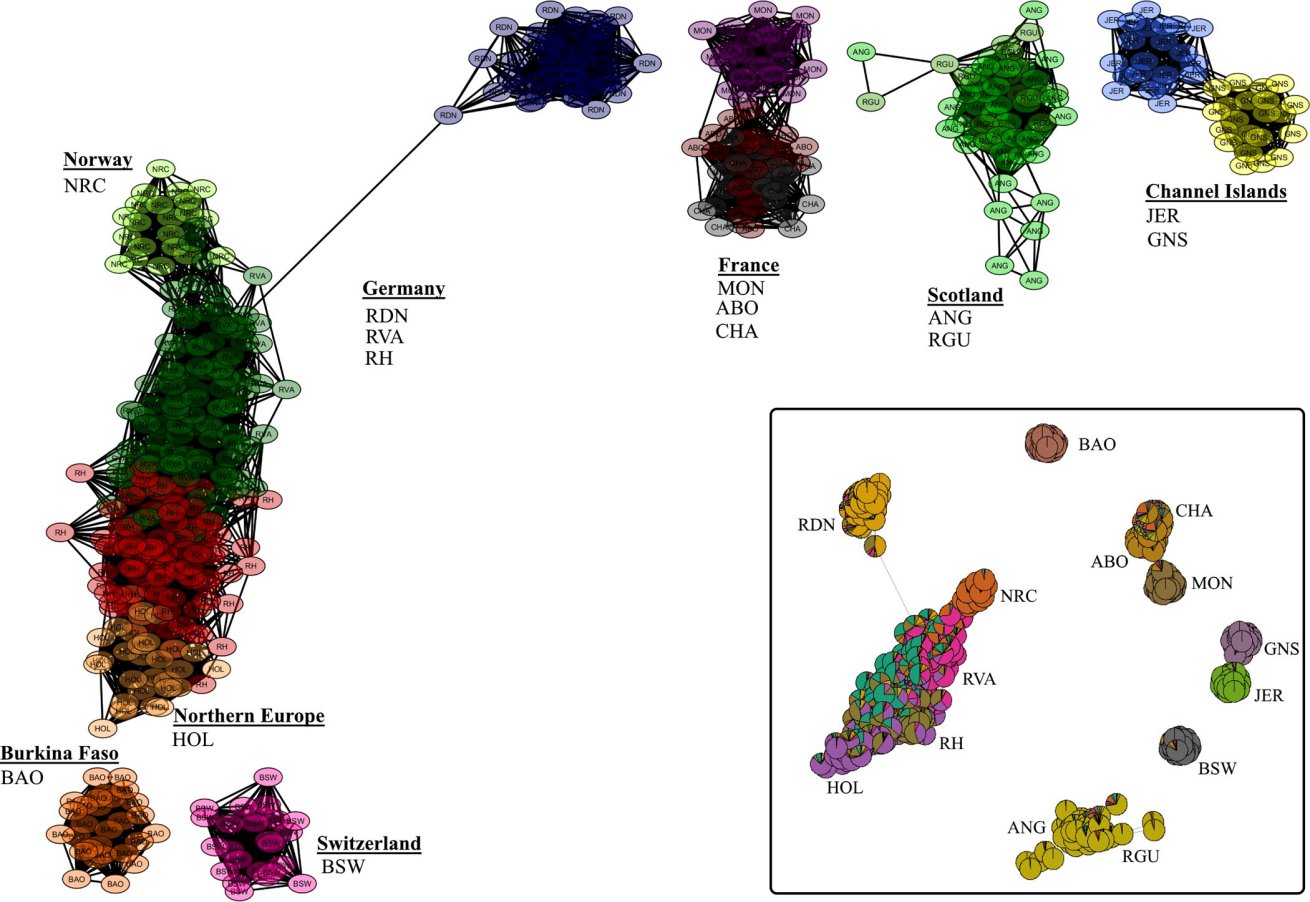
Network visualisation of 14 cattle breeds effectively separated along geographical lines with K-NN = 35. Admixture results at k = 19 were included in the network construction (in Box) such that node colouration reflects individual level of admixture.

The network visualization analysis provided a fine-tuned resolution of connectedness within and between breeds. In Fig 7, network graphics are presented for K-NN = 10 (A) and K-NN = 100 (B). At a K-NN value of 10, there exists a massive interconnection of networks between the Northern European breeds (HOL, RH, RVA and NRC). This, however, excluded RDN that formed a separate and single cluster. Samples of the ANG and RGU were also highly connected although a displacement of four ANG individual was observed. All the remaining breeds formed separate clusters and for ABO, BAO, GNS and NRC at least one individual was displaced from the breed cluster. Using a K-NN value of 100, the Channel Island breeds formed a single cluster that loosely connects to the rest of the European breeds. Consistent with both the PCA and Admixture results, there was no connection between BAO and the European breeds even at this high K-NN value. The use of an intermediate number of nearest neighbours (i.e. K-NN = 35) resulted in the formation of clusters and networks completely based of geographical origin (Fig 8). Notably, RDN was only loosely connected to the rest of the Northern breeds. By including the results of admixture analysis at the optimal number of k = 19 clusters, the proportions of genetic background of each sample (illustrated with node colours), alongside the sample’s connectedness with other genotypes are displayed (in Box). Generally, samples within a cluster had similar ancestry and for most breeds this tend to be uniform overall, while those from RVA and RH were most diverse.

## Discussion

Knowledge of the genetic diversity within and between breeds is essential for management decisions that aim at breed preservation. This is especially true for local breeds that are often small in numbers due to deliberate use of high yielding breeds in most commercial settings. The primary objective of this study was to assess whole-genome diversity of two local cattle (RVA and RDN) and a high yielding breed (RH) and subsequently, investigate patterns of admixture of these breeds applying different methods. Our study represents the first investigation of heterozygosity, molecular based *N*_*e*_ and ROH in RVA and RDN. Besides, admixture among the three breeds has never been reported.

### Genetic diversity and demographic changes

Average genomic relationship within breed was similar (0.031 vs. 0.033) for RVA and RDN and slightly lower (0.028) for RH. These estimates are much lower than the estimates reported by Signer-Hasler *et al*. [17] (0.044 to 0.155) for several cattle breeds. Among the breeds in the previous study [17], average genomic relationship for RH was 0.060. The sampling of individuals for a study can influence the overall genomic relationship among chosen animals. The data used in our study were already available and to mimic a randomly sampled population, a cap of 0.3 was put on the genomic relationship between any pair of individuals within a breed, i.e., following Burren *et al*. [22]. This could partly explain the observance of lower estimates of average genomic relationship in our study. Thus, the sampling of whole families for instance, could result in an overestimation of relatedness within populations. Observed heterozygosity has been used as a measure of within breed genetic diversity in several studies [15–17] in which analyses were essentially based on the Illumina BovineSNP50 BeadChip. Estimates of H_o_ in this study (0.356 to 0.374) fall within the range of values found in the previous studies: 0.357 to 0.399 in nine Swiss dairy cattle populations [17], 0.297 to 0.358 in thirty-two Italian cattle breeds [15] and 0.026 to 0.33 in fifty-three cattle breeds from around the world [16]. In the latter study [16], very low genetic diversity estimates (below 0.20) were found for some breeds, especially those from outside Europe. The authors attributed the differences in estimates to population demographic dynamics and to ascertainment bias [31,32] that tends to favour breeds that were included in the discovery of the BovineSNP50 chip assay. Similar to most European breeds, all the breeds in our study had a high estimate of heterozygosity. The H_o_ estimates are consistent with estimates of *N*_*e*_ in most recent generations. From our results, RVA maintained the highest genetic diversity among the breeds with an estimate of Ne_5Gen_ above 100. Smaller *N*_*e*_ estimates in RDN (67) and RH (86) may suggest limited gene flow from other breeds during the developmental history of these breeds. Trends in *N*_*e*_ however, revealed larger estimates for all breeds in earlier generations. Reduction in *N*_*e*_ across generations in this study suggests the occurrence of a past genetic bottleneck in all breeds and this is consistent with findings of many other studies [3,15,43,44]. The 2018 annual report on cattle production in Germany provided census numbers including 595,929 (RH), 110,000 (RDN) and 30,411 (RVA) [45], however, the number of herdbook animals is much lower. Thus, the census numbers can be deceptive, as they do not accurately inform of the genetic diversity present in the breeds. RVA has a smaller census number; nevertheless, it maintained the highest genetic diversity probably due to high levels of introgression activity. This is to some extent supported by the observed trend in LD decay. RVA showed a faster LD decay indicative of high within breed diversity. In RDN and RH, decay was slower probably due to the presence of long haplotypes. Therefore, average inbreeding is expected to be higher in these latter breeds.

### Usefulness of ROH inbreeding

Wright’s [46] pedigree inbreeding concept has a long history of widespread and routine use in genetics. Pedigree inbreeding estimation assumes a base population in which individuals are unrelated and non-inbred and it has the tendency to underestimate realised relationships. Nowadays, the increased availability of cost effective genomic data in the form of SNPs has made it possible to develop methods for the estimation of genomic inbreeding. In a previous study, F_ROH_ and F_HOM_ were found to be more precise and often less biased than F_PED_ [47]. Among the genomic estimators, F_ROH_ presents some advantages that can make it the method of choice. For instance, ROH length can be used to distinguish between recent and ancient inbreeding. Thus, long ROH are indicative of recent inbreeding whereas short ROH predict inbreeding of ancient origin [38,47]. F_ROH_ is also not affected by allele frequencies [48] and it only takes values between zero (0) and one (1). Consequently, it provides the best premise for comparison with F_PED_. ROH detection parameters in this study were somewhat adapted to the thresholds used by Purfield *et al*. [49] for the Bovine SNP50 panel in one of the most extensive studies of ROH in cattle. For all our breeds, the majority of ROH were observed in the length class >5 to 10 Mb. This is consistent with the finding of Signer-Hasler *et al*. [17] but contrary to other findings [49]. In the latter, Purfield *et al*. [49] found ROH of length class 1 to 5 Mb to be more frequent in using the high density SNP panel (777,972 SNPs). Our observation of very few ROHs (less than 4%) in the length class 1 to 5 Mb may be an inherent property of the Illumina BovineSNP50 BeadChip. Validating the panel’s reliability to infer ROH that had been previously detected with greater accuracy using HD array, Purfield *et al*. [49] reported a failure of the reduced panel to detect about 27% of ROH in the length class 1 to 5 Mb. However, the proportion of ROH in this length class reported by Signer-Hasler *et al*. [17] was still high (about 18 to 35%) though the overall distribution was similar to what we found. In fact, all ROH detected in our study were above 4 Mb even when no restriction was put on the minimum ROH length. Hence, it is not surprising to have the smallest proportion of ROH in the length class 1 to 5 Mb. Undoubtedly, old inbreeding contributed to overall inbreeding in our breeds, however, a substantial proportion of this contribution arose from ancestors in generations not too distant in time, probably around 12 generations ago under the assumption that 1 cM = 1 Mb. Thus, inbreeding coefficients calculated from ROH with minimum length higher than 4, 8 and 16 Mb are expected to correspond to the reference ancestral population being remote approximately 12, 6 and 3 generations, respectively [37]. This actually forms the basis for calculating F_ROH>4_, F_ROH>8_ and F_ROH>16_ in this study. The estimated F_ROH>16_ showed that recent inbreeding is more a problem in RDN and RH than in RVA. This is not different for the other minimum ROH length measures. These finding are consistent with the H_o_ and *N*_*e*_ estimates reported. Red Holstein is a high yielding breed whose performance is next to the Black-and-white Holstein in the region (Schleswig-Holstein). Average milk yield of the breed recorded in 2017 was 8,208 kg compared to that of RVA (7,638 kg) and RDN (6,738 kg) [45]. Use of reproductive techniques such as sexed semen in first inseminations is more intense and has a much longer history in RH [45]. Such techniques promote wide spread use of few animals with the consequence of increased inbreeding levels.

Negative average values of F_HOM_ were found for all our breeds and this was not surprising since the estimation procedure made use of the current sample of individual as reference [50]. Purcell *et al*. [13] warned that strongly negative values of this parameter could stem from sample contamination events. However, Wang [50] explained that such negative values can be legitimate and must not be interpreted as probabilities, rather, the expected relative deficit of the occurrences of homologous genes that are identical in state within individuals due to the relative deficit of shared ancestry. Inbreeding estimates based on the pedigree method were found to be generally lower than those from F_ROH_ and this is also the case for many other studies in cattle [14,17,48]. Similar to our finding, the magnitude of the difference between F_ROH_ and F_PED_ was about a double in Zhang *et al*.*’s* study [48] when a reduced SNP panel was used and even larger in using sequenced data. In Signer-Hasler *et al*.’s study [17] however, only a slight difference was observed between the two inbreeding estimators, for instance, estimates of 0.045 and 0.042 were obtained for F_ROH_ and F_PED_, respectively, in RH. Lower estimates of F_PED_ can be the results of incomplete and shallow-depth pedigree. In the current study, average pedigree completeness at the fifth parental generation was 88% for RVA and 89% for RDN. At that same parental generation in Signer-Hasler *et al*.’s study [17], pedigree completeness was above 99% for all breeds whose F_PED_ estimates did not deviate much from the estimates of F_ROH._ It is not surprising that the authors found an extremely large difference between the two estimates for the breed (Evolèner) that had a pedigree completeness of 36%. Nevertheless, our regression analysis revealed a linear and positive relationship between all the inbreeding estimators. F_ROH>4_ tend to correlate highly with F_PED_ and F_HOM_ than did either *F*_ROH>8_ or *F*_ROH>16_. Notice is to be made that F_ROH>4_ is equivalent to a no restriction on minimum ROH length (i.e. F_ROH>4_ = F_ROH_) in this study. Our correlation estimates (0.701, F_ROH_, F_PED_; 0.752, F_ROH_, F_HOM_ and 0.511, F_HOM_, F_PED_) were in the range of estimates reported in previous studies [14,17,49,51,52] for cattle. Signer-Hasler *et al*. [17] reported values of 0.701 (F_ROH_, F_PED_) and 0.677 (F_HOM_, F_PED_) in using pedigrees with average completeness of at least 95%. In humans genetic studies, a higher value (*r*_*p*_ = 0.86) was reported between F_ROH_ and F_PED_ when the minimum length of ROH was restricted to 1.5 Mb [28]. In Swiss goat breeds, Burren *et al*. [22] also reported correlation coefficient of 0.808 between F_ROH_ and F_PED_ from the use of pedigree information subjected to routine parentage testing. Meanwhile, the intercept of the regression line revealed a real advantage of F_ROH_ over F_PED_. When F_ROH>16_ was regressed on F_PED_, the intercept was close to zero (0.002). Regressing F_ROH>4_ and F_ROH>8_ on F_PED_ on the other hand resulted in higher estimates of intercept (i.e. 0.011 and 0.008, respectively). Recalling that shorter ROH predict ancient inbreeding, F_ROH>4_ captured information on past inbreeding that was neither detected by F_ROH>16_ nor by F_PED_. Similar findings were reported for analyses involving several cattle breeds [49] and also in human genetic studies [28].

### Population structure

Based on 19,971 autosomal SNPs, all the three measures of population structure analysis effectively separated the 14 breeds based on their geographical origin and this is important in validating the results of our study. Further differentiation as was observed for 1) JER and GNS, 2) MON, CHA and ABO, 3) NRC and HOL and 4) ANG and RGU at higher k values of admixture are consistent with findings of a previous study [1] from which outgroup data were obtained. One of the aims of the current study was to investigate admixture and gene flow events amongst RVA, RH and RDN. Our study provided genomic evidence of common genetic background between these breeds. Variation, however, existed in the degree of ancestor sharing amongst the breeds. RVA hugely resemble RH at the optimal level of k (Fig 6) and it harboured a fraction of the unique background (green-yellow) from the Norwegian breed. The latter suggests an introgression of RVA with NRC genetics and although this is also true for both PCA and NetView findings, no such report exist to our knowledge. There has been the mentioning of introgressive hybridization to improve the performance of RVA [7] and RDN [8] in previous studies. RVA is known to have been crossed with Danish Red cattle to improve body size and milk yield around 1928 and with Red Holstein, Swedish and Finnish cattle after the 1960s [53]. According to Bennewitz and Meuwissen [7], the original RVA breed is close to final extinction. In RDN, Andresen *et al*. [8] mentioned the harbouring of up to 25% RH genes in the breed’s pedigree. Compared to RVA, the proximity of RDN to RH seemed quite distant (Figs 5 and 7). However, even at high k values of the admixture analysis, low levels of introgression from RH could be detected in RDN. Apparently, studies that investigate relatedness between our breeds are lacking. The one study available [10], reported a separation (though somewhat close) of RVA and RH into different clusters using principal component analysis. In the same study, however, a high kinship coefficient value was found between the two breeds.

The NetView results supported the Admixture and PCA findings and provided deeper insight into the interconnectedness within and between breeds. The choice of K-NN value is an important factor that influences the topological structure of network graphs [12]. A K-NN value of 10 was suggested and successfully used in previous studies [24,26,27,54]. Usually, larger values of K-NN aid the investigation of admixture between populations whereas smaller values of the parameter investigates fine-scale genetic substructure. Thus, a K-NN value of 10 has been shown to be effective in detecting both large- and fine-scale genetic structures [42]. In the current study, the use of K-NN value of 10 separated most of the breeds into individual breed clusters except those that had high level of resemblance (Fig 7A). Additionally, individuals that tended to show some level of dissimilarity to the breed group were distinguished. K-NN = 35 (Fig 8) elucidated regional differences while K = 100 (Fig 7B) provided insight into continental breed differences. By including the results of Admixture at the optimal level of k in the network visualisation analysis, the level of admixture as indicated by node colour (Fig 8, Box) was highest in RVA and RH. This corroborates the admixture analysis at k = 19. For RVA, a highly mixed genetic background may reflects an artificial increase in heterozygosity consistent with the breed’s history and large effective population size.

## Conclusion

In this study, all three measures of population structure analysis confirmed high levels of introgressive gene flow from RH to RVA. These breeds also showed highly heterogeneous genetic background. The study for the first time uncovered the presence of Norwegian Red cattle genes in RVA suggestive of past gene flow event between the two breeds. Introgression of RDN with RH genes was comparatively limited and detectable only through Admixture analysis. Unlike the use of herdbook number of male and female animals for the determination of endangerment status as in the case of our breeds of interest, genomic measures of diversity provide a powerful premise beyond the limitations of pedigree analysis on which basis conservation decisions can be made. The study emphasised the advantages of ROH inbreeding over pedigree inbreeding and showed the highest level of genetic diversity in RVA. Evidently, the inbreeding levels in our breeds were low and did not raise alarming concerns about the within breed genetic diversity. However, the constantly decreasing effective population size across generations should inform decisions on conservation strategies for a proper management of the breeds.

## Supporting information

S1 Table Description of the number of informative SNPs, length covered by SNPs, average, minimum and maximum distances and average r^2^ (linkage disequilibrium) between adjacent markers of the 29 autosomes. (DOCX)

S1 Fig Completeness of the Angler (RVA) and Red-and-white dual-purpose (RDN) cattle pedigrees across parental generations. (TIF)

S2 Fig Regression plot of F_HOM_ on F_PED_ for the Angler (RVA) and Red-and-White dual-purpose (RDN) breeds. The broken line (black) is a regression line for all breeds and corresponds to the regression equation presented.
(TIF)

S3 Fig Development of the cross‐validation for different number of clusters (k). Cross validation error was lowest for k = 19, which indicates an optimal number of 19 clusters.
(TIF)

S4 Fig Admixture graphs for k ranging from 2 to 20, with the optimal cluster level marked with an arrow. The amount of a colour in a cluster reflects a breed’s proportion of genetic variation originating from that colour. Breed names are coloured according to geographical origin: Germany (red), Northern Europe (grey), Norway (green), Channel Islands (dark red), Switzerland (blue), France (purple), Scotland (brown) and Burkina Faso (orange).
(TIF)

## References

[1] Gautier M, Laloë D, Moazami-Goudarzi K. Insights into the genetic history of French cattle from dense SNP data on 47 worldwide breeds. PLoS One. 2010;5: e13038 10.1371/journal.pone.0013038 20927341 PMC2948016

[2] Ajmone-Marsan P, Garcia JF, Lenstra JA. On the origin of cattle: How aurochs became cattle and colonized the world. Evol Anthropol. 2010;19: 148–157. 10.1002/evan.20267

[3] Kukučková V, Moravčíková N, Ferenčaković M, Simčič M, Mészáros G, Sölkner J, et al Genomic characterization of Pinzgau cattle: genetic conservation and breeding perspectives. Conserv Genet. 2017;18: 893–910. 10.1007/s10592-017-0935-9

[4] Felius M, Beerling M-L, Buchanan DS, Theunissen B, Koolmees PA, Lenstra JA. On the history of cattle genetic resources. Diversity. 2014;6: 705–750. 10.3390/d6040705

[5] Adamczyk K, Felenczak A, Jamrozy J, Szarek J, Bulla J. Conservation of Polish Red cattle. Slovak J Anim Sci. 2008;41: 72–76.

[6] Bay E, Colinet F, Gengler N. Dual Purpose Red and White [Internet]. EU GENRES 870/04; 2010 Available: http://www.regionalcattlebreeds.eu/wp/documents/WP1-Breedcase-DP-RedandWhite.pdf

[7] Bennewitz J, Meuwissen THE. Estimation of extinction probabilities of five German cattle breeds by population viability analysis J Dairy Sci. Elsevier; 2005;88: 2949–2961. 10.3168/jds.S0022-0302(05)72975-1 16027209

[8] Andresen U, Bartjen A, Kaske M. German Red and White Coloured Dual Purpose an alternative for a sustainable milk production? Tierarztl Umsch. 2014;69: 537–542.

[9] Addo S, Schäler J, Hinrichs D, Thaller G. Genetic Diversity and Ancestral History of the German Angler and the Red-and-White Dual-Purpose Cattle Breeds Assessed through Pedigree Analysis. Agric Sci. 2017;8: 1033–1047.

[10] Wang Y, Segelke D, Emmerling R, Bennewitz J, Wellmann R. Long-Term Impact of Optimum Contribution Selection Strategies on Local Livestock Breeds with Historical Introgression Using the Example of German Angler Cattle. G3 (Bethesda). 2017;7: 4009–4018. 10.1534/g3.117.300272 29089375 PMC5714497

[11] Kawęcka A, Gurgul A, Miksza-Cybulska A. The Use of SNP Microarrays for Biodiversity Studies of Sheep–A Review. Ann Anim Sci. De Gruyter Open; 2016;16: 975–987. 10.1515/aoas-2016-0017

[12] Lenstra JA, Groeneveld LF, Eding H, Kantanen J, Williams JL, Taberlet P, et al Molecular tools and analytical approaches for the characterization of farm animal genetic diversity. Anim Genet. 2012;43: 483–502. 10.1111/j.1365-2052.2011.02309.x 22497351

[13] LaFramboise T. Single nucleotide polymorphism arrays: a decade of biological, computational and technological advances. Nucleic Acids Res. 2009;37: 4181–4193. 10.1093/nar/gkp552 19570852 PMC2715261

[14] Forutan M, Mahyari SA, Baes C, Melzer N, Schenkel FS, Sargolzaei M. Inbreeding and runs of homozygosity before and after genomic selection in North American Holstein cattle. BMC Genomics. 2018;19: 98 10.1186/s12864-018-4453-z 29374456 PMC5787230

[15] Mastrangelo S, Ciani E, Marsan PA, Bagnato A, Battaglini L, Bozzi R, et al Conservation status and historical relatedness of Italian cattle breeds. Genet Sel Evol. 2018;50: 35 10.1186/s12711-018-0406-x 29940848 PMC6019226

[16] Orozco-terWengel P, Barbato M, Nicolazzi E, Biscarini F, Milanesi M, Davies W, et al Revisiting demographic processes in cattle with genome-wide population genetic analysis. Front Genet. 2015;6: 191 10.3389/fgene.2015.00191 26082794 PMC4451420

[17] Signer-Hasler H, Burren A, Neuditschko M, Frischknecht M, Garrick D, Stricker C, et al Population structure and genomic inbreeding in nine Swiss dairy cattle populations. Genet Sel Evol. 2017;49: 83 10.1186/s12711-017-0358-6 29115934 PMC5674839

[18] Purfield DC, McParland S, Wall E, Berry DP. The distribution of runs of homozygosity and selection signatures in six commercial meat sheep breeds. PLoS One. 2017;12: e0176780 Available: 10.1371/journal.pone.0176780 28463982 PMC5413029

[19] Deniskova TE, Dotsev A V, Selionova MI, Kunz E, Medugorac I, Reyer H, et al Population structure and genetic diversity of 25 Russian sheep breeds based on whole-genome genotyping. Genet Sel Evol. 2018;50: 29 10.1186/s12711-018-0399-5 29793424 PMC5968526

[20] Edea Z, Dessie T, Dadi H, Do K-T, Kim K-S. Genetic Diversity and Population Structure of Ethiopian Sheep Populations Revealed by High-Density SNP Markers. Front Genet. 2017;8: 218 Available: 10.3389/fgene.2017.00218 29312441 PMC5744078

[21] Manunza A, Noce A, Serradilla JM, Goyache F, Martínez A, Capote J, et al A genome-wide perspective about the diversity and demographic history of seven Spanish goat breeds. Genet Sel Evol. 2016;48: 52 10.1186/s12711-016-0229-6 27455838 PMC4960707

[22] Burren A, Neuditschko M, Signer‐Hasler H, Frischknecht M, Reber I, Menzi F, et al Genetic diversity analyses reveal first insights into breed-specific selection signatures within Swiss goat breeds. Anim Genet. 2016;47: 727–739. 10.1111/age.12476 27436146

[23] Grilz-Seger G, Mesarič M, Cotman M, Neuditschko M, Druml T, Brem G. Runs of Homozygosity and Population History of Three Horse Breeds With Small Population Size. J Equine Vet Sci. 2018;71: 27–34. 10.1016/j.jevs.2018.09.004

[24] Druml T, Neuditschko M, Grilz-Seger G, Horna M, Ricard A, Mesarič M, et al Population networks associated with runs of homozygosity reveal new insights into the breeding history of the Haflinger horse. J Hered. 2017;109: 384–392. 10.1093/jhered/esx114 29294044

[25] Alexander DH, Novembre J, Lange K. Fast model-based estimation of ancestry in unrelated individuals. Genome Res. 2009;19: 1655–64. 10.1101/gr.094052.109 19648217 PMC2752134

[26] Neuditschko M, Khatkar MS, Raadsma HW. NetView: a high-definition network-visualization approach to detect fine-scale population structures from genome-wide patterns of variation. PLoS One. 2012;7: e48375 10.1371/journal.pone.0048375 23152744 PMC3485224

[27] Steinig EJ, Neuditschko M, Khatkar MS, Raadsma HW, Zenger KR. netview p: a network visualization tool to unravel complex population structure using genome‐wide SNPs. Mol Ecol Resour. 2015;16: 216–227. 10.1111/1755-0998.12442 26129944

[28] McQuillan R, Leutenegger A-L, Abdel-Rahman R, Franklin CS, Pericic M, Barac-Lauc L, et al Runs of homozygosity in European populations. Am J Hum Genet. 2008;83: 359–372. 10.1016/j.ajhg.2008.08.007 18760389 PMC2556426

[29] Purcell S, Neale B, Todd-Brown K, Thomas L, Ferreira MAR, Bender D, et al PLINK: a tool set for whole-genome association and population-based linkage analyses. Am J Hum Genet. 2007;81: 559–575. 10.1086/519795 17701901 PMC1950838

[30] Sempéré G, Moazami-Goudarzi K, Eggen A, Laloë D, Gautier M, Flori L. WIDDE: a Web-Interfaced next generation database for genetic diversity exploration, with a first application in cattle. BMC Genomics. BioMed Central; 2015;16: 940. 10.1186/s12864-015-2181-1 PMC4647285 26573482

[31] Gautier M, Flori L, Riebler A, Jaffrézic F, Laloé D, Gut I, et al A whole genome Bayesian scan for adaptive genetic divergence in West African cattle. BMC Genomics. 2009;10: 550 10.1186/1471-2164-10-550 19930592 PMC2784811

[32] Matukumalli LK, Lawley CT, Schnabel RD, Taylor JF, Allan MF, Heaton MP, et al Development and characterization of a high density SNP genotyping assay for cattle. PLoS One. 2009;4: e5350 10.1371/journal.pone.0005350 19390634 PMC2669730

[33] Barbato M, Orozco-terWengel P, Tapio M, Bruford MW. SNeP: a tool to estimate trends in recent effective population size trajectories using genome-wide SNP data. Front Genet. 2015;6: 109 Available: https://www.frontiersin.org/article/10.3389/fgene.2015.00109 25852748 10.3389/fgene.2015.00109 PMC4367434

[34] Corbin LJ, Liu AYH, Bishop SC, Woolliams JA. Estimation of historical effective population size using linkage disequilibria with marker data. J Anim Breed Genet. 2012;129: 257–270. 10.1111/j.1439-0388.2012.01003.x 22775258

[35] Hayes BJ, Visscher PM, McPartlan HC, Goddard ME. Novel multilocus measure of linkage disequilibrium to estimate past effective population size. Genome Res. 2003;13: 635–643. 10.1101/gr.387103 12654718 PMC430161

[36] Sved JA, Feldman MW. Correlation and probability methods for one and two loci. Theor Popul Biol. 1973;4: 129–132. 10.1016/0040-5809(73)90008-7 4726005

[37] Mészáros G, Boison SA, Pérez O’Brien AM, Ferenčaković M, Curik I, Da Silva MVB, et al Genomic analysis for managing small and endangered populations: a case study in Tyrol Grey cattle. Front Genet. 2015;6: 173 10.3389/fgene.2015.00173 26074948 PMC4443735

[38] Keller MC, Visscher PM, Goddard ME. Quantification of inbreeding due to distant ancestors and its detection using dense SNP data. Genetics. 2011;189: 237–249. 10.1534/genetics.111.130922 21705750 PMC3176119

[39] Meuwissen THE, Luo Z. Computing inbreeding coefficients in large populations. Genet Sel Evol. 1992;24: 305 10.1186/1297-9686-24-4-305

[40] Boichard D. PEDIG: a fortran package for pedigree analysis suited for large populations. Proc 7th world Congr Genet Appl to Livest Prod. 2002;32: 525–528.

[41] Francis RM. POPHELPER: an R package and web app to analyse and visualize population structure. Mol Ecol Resour. 2017;17: 27–32. 10.1111/1755-0998.12509 26850166

[42] Shannon P, Markiel A, Ozier O, Baliga NS, Wang JT, Ramage D, et al Cytoscape: a software environment for integrated models of biomolecular interaction networks Genome Res. Cold Spring Harbor Lab; 2003;13: 2498–2504. 10.1101/gr.1239303 14597658 PMC403769

[43] Iso‐Touru T, Tapio M, Vilkki J, Kiseleva T, Ammosov I, Ivanova Z, et al Genetic diversity and genomic signatures of selection among cattle breeds from Siberia, eastern and northern Europe. Anim Genet. 2016;47: 647–657. 10.1111/age.12473 27629771

[44] Yurchenko A, Yudin N, Aitnazarov R, Plyusnina A, Brukhin V, Soloshenko V, et al Genome-wide genotyping uncovers genetic profiles and history of the Russian cattle breeds. Heredity (Edinb). 2018;120: 125–137. 29217829 10.1038/s41437-017-0024-3 PMC5837115

[45] Arbeitsgemeinschaft Deutscher Rinderzüchter. Rinderproduktion in Deutschland 2017 Arbeitsgemeinschaft Deutscher Rinderzüchter e.V. (ADR), Bonn, Germany; 2018.

[46] Wright S. Coefficients of inbreeding and relationship. Amer Nat. 1922;56 10.1086/279872

[47] Kardos M, Luikart G, Allendorf FW. Measuring individual inbreeding in the age of genomics: marker-based measures are better than pedigrees. Heredity (Edinb). 2015;115: 63. 26059970 10.1038/hdy.2015.17 PMC4815495

[48] Zhang Q, Calus MP, Guldbrandtsen B, Lund MS, Sahana G. Estimation of inbreeding using pedigree, 50k SNP chip genotypes and full sequence data in three cattle breeds. BMC Genet. 2015;16 10.1186/s12863-015-0168-1 26195126 PMC4509611

[49] Purfield DC, Berry DP, McParland S, Bradley DG. Runs of homozygosity and population history in cattle. BMC Genet. 2012;13: 70 10.1186/1471-2156-13-70 22888858 PMC3502433

[50] Wang J. Marker-based estimates of relatedness and inbreeding coefficients: an assessment of current methods. J Evol Biol. 2014;27: 518–530. 10.1111/jeb.12315 24444019

[51] Marras G, Gaspa G, Sorbolini S, Dimauro C, Ajmone-Marsan P, Valentini A, et al Analysis of runs of homozygosity and their relationship with inbreeding in five cattle breeds farmed in Italy. Anim Genet. 2015;46 10.1111/age.12259 25530322

[52] Ferenčaković M, Hamzić E, Gredler B, Solberg TR, Klemetsdal G, Curik I, et al Estimates of autozygosity derived from runs of homozygosity: empirical evidence from selected cattle populations. J Anim Breed Genet. 2013;130: 286–293. 10.1111/jbg.12012 23855630

[53] Porter V, Alderson L, Hall SJG, Sponenberg DP. Mason’s World Encyclopedia of Livestock Breeds and Breeding: 2 Volume Pack 6th ed Boston: Cabi; 2016.

[54] Burren A, Signer-Hasler H, Neuditschko M, Tetens J, Kijas J, Drögemüller C, et al Fine-scale population structure analysis of seven local Swiss sheep breeds using genome-wide SNP data. Anim Genet Resour. 2014;55: 67–76.

