# Supplementary material for: Runs of Homozygosity and NetView analyses provide new insight into the genome-wide diversity and admixture of three German cattle breeds
Source: PLoS One. 2019 Dec 4;14(12):e0225847. doi: 10.1371/journal.pone.0225847 (PMC6892555; doi:10.1371/journal.pone.0225847)
Supplement: S1 Table — (DOCX) [file pone.0225847.s001.docx]

**S1Table. Description of the number of informative SNPs, length covered by SNPs, average, minimum and maximum distances and average r^2^ (linkage disequilibrium) between adjacent markers of the 29 autosomes.**

| Chr | Number of SNPs | Length (kb) | Average distance (kb) | Minimum distance (kb) | | Maximum distance (kb) | Average r^2^ |
| --- | --- | --- | --- | --- | --- | --- | --- |
| 1 | 2695 | 158094.120 | 58.684 | 0.131 | 516.643 | | 0.209 |
| 2 | 2160 | 136662.110 | 63.299 | 0.075 | 597.921 | | 0.201 |
| 3 | 2001 | 121144.180 | 60.572 | 0.108 | 840.964 | | 0.205 |
| 4 | 1981 | 120598.157 | 60.908 | 0.365 | 378.555 | | 0.196 |
| 5 | 1715 | 121078.748 | 69.948 | 0.104 | 663.266 | | 0.190 |
| 6 | 2054 | 119193.568 | 57.306 | 2.66 | 690.926 | | 0.211 |
| 7 | 1788 | 112384.068 | 61.358 | 1.969 | 747.762 | | 0.201 |
| 8 | 1872 | 112861.845 | 60.322 | 0.084 | 481.649 | | 0.195 |
| 9 | 1597 | 105464.416 | 66.080 | 0.449 | 666.907 | | 0.192 |
| 10 | 1728 | 103180.360 | 57.794 | 0.284 | 619.620 | | 0.193 |
| 11 | 1769 | 107136.657 | 60.598 | 4.786 | 833.187 | | 0.189 |
| 12 | 1342 | 90944.479 | 63.757 | 0.237 | 842.603 | | 0.180 |
| 13 | 1412 | 83835.601 | 59.416 | 0.065 | 615.150 | | 0.202 |
| 14 | 1412 | 83152.514 | 58.232 | 0.108 | 669.778 | | 0.218 |
| 15 | 1318 | 84221.946 | 63.950 | 2.924 | 851.292 | | 0.176 |
| 16 | 1321 | 81249.445 | 60.648 | 0.178 | 693.304 | | 0.187 |
| 17 | 1263 | 74887.030 | 59.340 | 2.032 | 813.742 | | 0.173 |
| 18 | 1049 | 65401.137 | 61.409 | 5.603 | 485.718 | | 0.197 |
| 19 | 1093 | 63513.847 | 58.163 | 1.014 | 937.787 | | 0.165 |
| 20 | 1270 | 71594.660 | 56.418 | 1.527 | 559.097 | | 0.183 |
| 21 | 1092 | 71097.978 | 64.014 | 0.903 | 763.726 | | 0.198 |
| 22 | 1005 | 61096.841 | 60.853 | 2.432 | 465.568 | | 0.183 |
| 23 | 845 | 52068.731 | 61.693 | 1.568 | 836.641 | | 0.147 |
| 24 | 1018 | 62101.915 | 61.064 | 0.095 | 594.958 | | 0.184 |
| 25 | 786 | 42712.739 | 54.411 | 0.784 | 290.523 | | 0.171 |
| 26 | 875 | 50953.355 | 58.299 | 0.281 | 394.539 | | 0.179 |
| 27 | 770 | 45332.323 | 57.219 | 0.151 | 534.557 | | 0.162 |
| 28 | 773 | 46153.667 | 59.785 | 0.675 | 399.563 | | 0.148 |
| 29 | 847 | 51102.335 | 58.855 | 1.860 | 494.291 | | 0.169 |
| Total | **40851** | **2,499,218.772** | **60.496** | **0.065** | **937.787** | | **0.186** |
