# Supplementary figures and images for: Runs of Homozygosity and NetView analyses provide new insight into the genome-wide diversity and admixture of three German cattle breeds
Source: PLoS One. 2019 Dec 4;14(12):e0225847. doi: 10.1371/journal.pone.0225847 (PMC6892555; doi:10.1371/journal.pone.0225847)

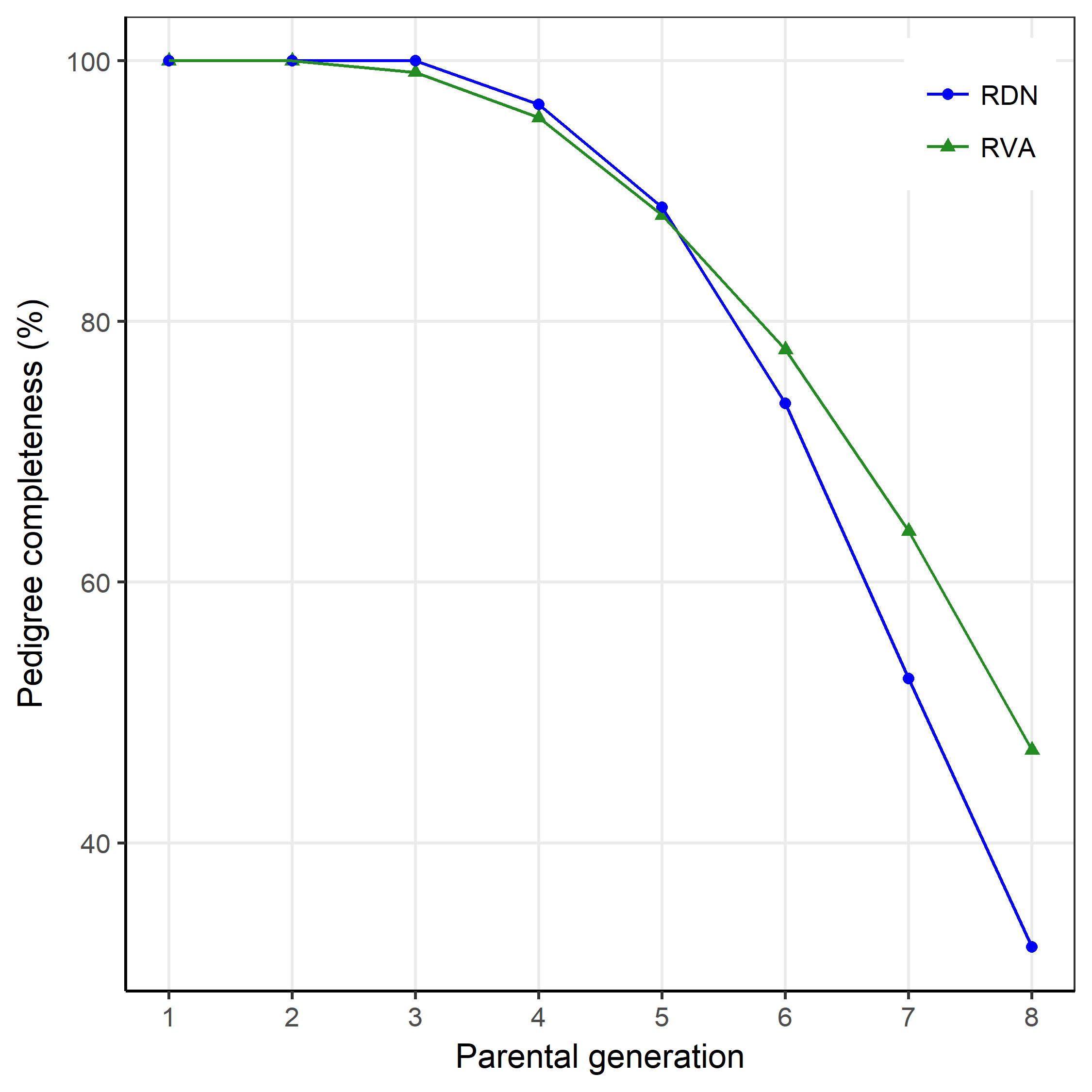

Supplement: S1 Fig — (TIF) [file pone.0225847.s002.tif]

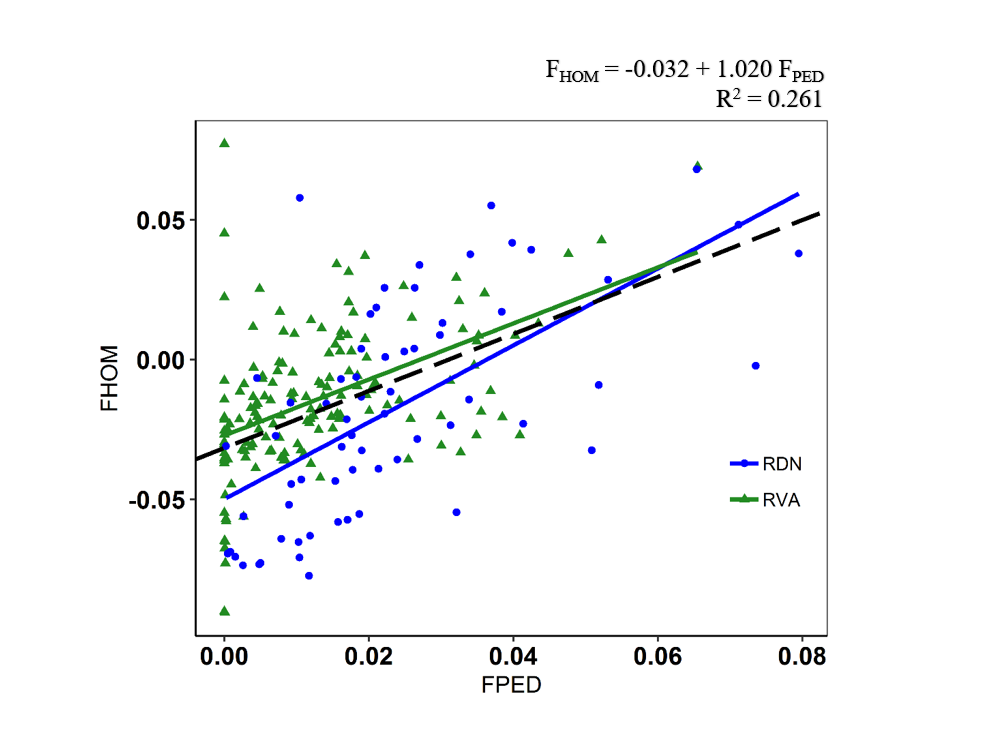

Supplement: S2 Fig — The broken line (black) is a regression line for all breeds and corresponds to the regression equation presented. (TIF) [file pone.0225847.s003.tif]

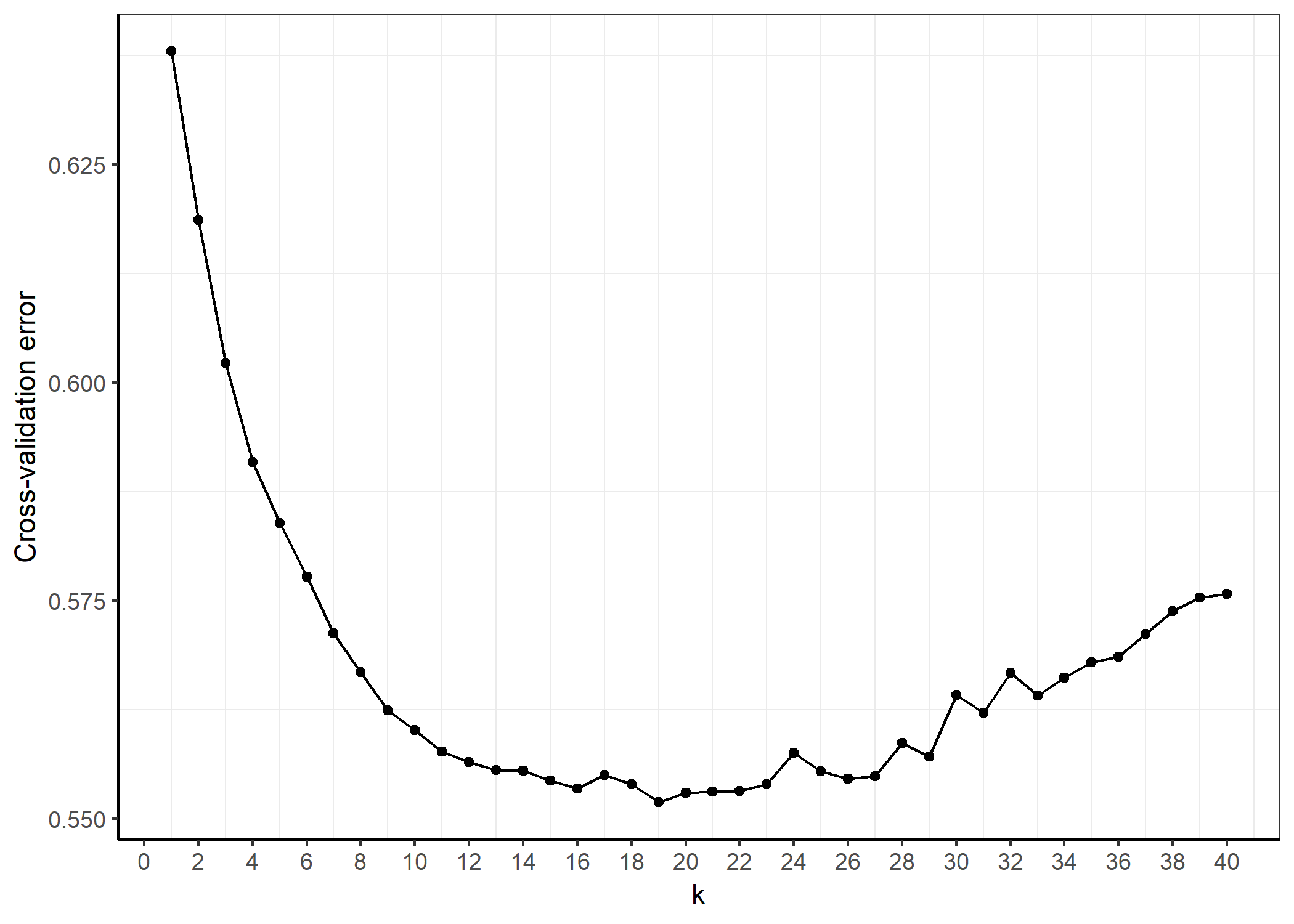

Supplement: S3 Fig — Cross validation error was lowest for k = 19, which indicates an optimal number of 19 clusters. (TIF) [file pone.0225847.s004.tif]

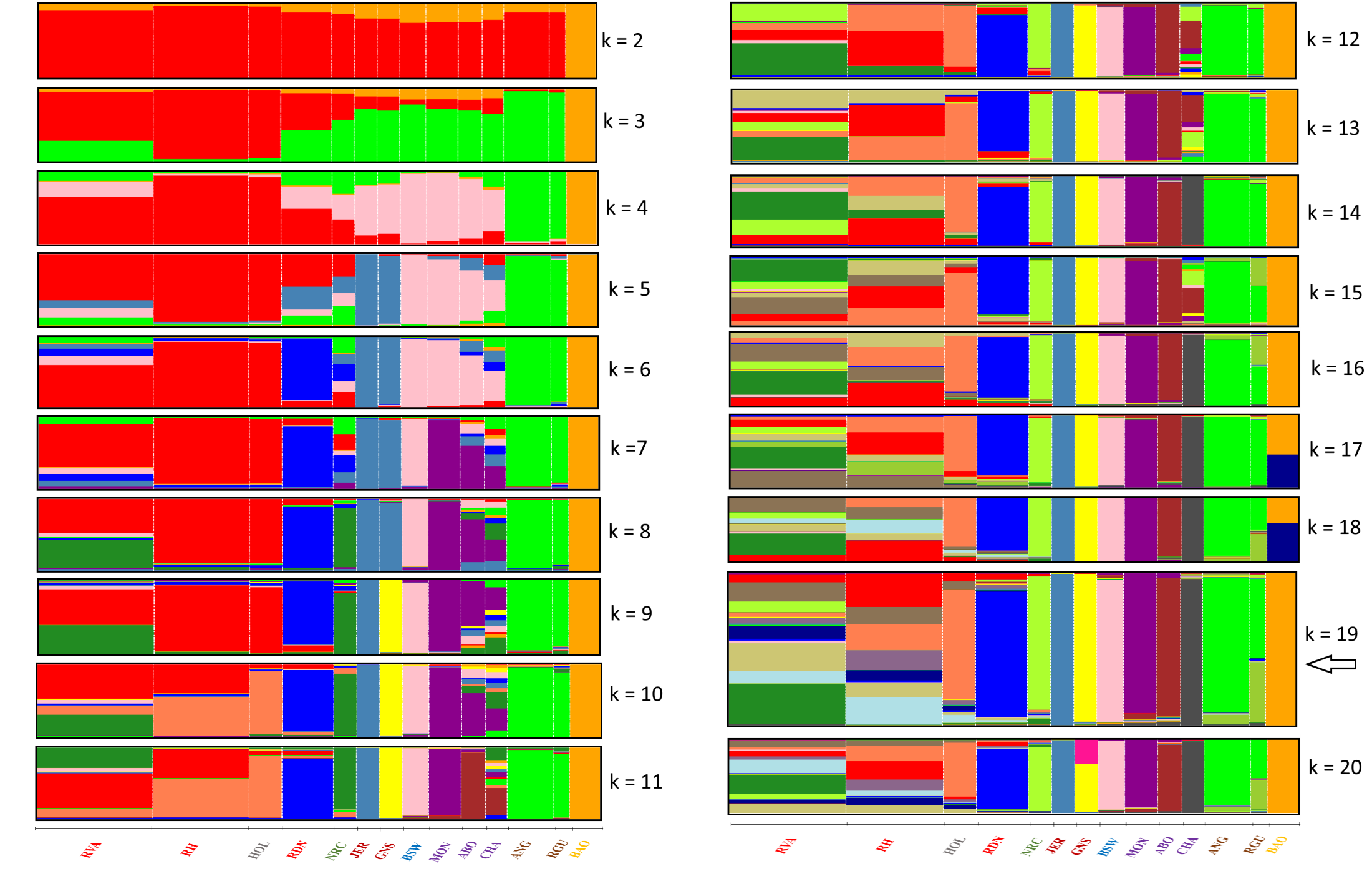

Supplement: S4 Fig — The amount of a colour in a cluster reflects a breed’s proportion of genetic variation originating from that colour. Breed names are coloured according to geographical origin: Germany (red), Northern Europe (grey), Norway (green), Channel Islands (dark red), Switzerland (blue), France (purple), Scotland (brown) and Burkina Faso (orange). (TIF) [file pone.0225847.s005.tif]
